# Bipolar plasma vaporization versus monopolar TUR and “cold-knife" TUI in secondary bladder neck sclerosis – An evidence based, retrospective critical comparison in a single center clinical setting

**Published:** 2014-03-25

**Authors:** C Moldoveanu, B Geavlete, M Jecu, F Stanescu, L Adou, C Bulai, C Ene, P Geavlete

**Affiliations:** "Saint John" Emergency Clinical Hospital, Department of Urology

**Keywords:** bipolar plasma vaporization, monopolar transurethral resection, “cold-knife" transurethral incision, secondary bladder neck sclerosis

## Abstract

Introduction: A long term, retrospective study was performed aiming to outline a critical comparison concerning the efficacy, safety and durability of the bipolar plasma vaporization (BPV), standard monopolar transurethral resection (TUR) and “cold-knife" “star" transurethral incision (TUI) in secondary bladder neck sclerosis (BNS) cases.

Materials & Methods: Of the 126 patients included in the trial based on maximum flow rate (Qmax) below 10 mL/s and International Prostate Symptom Score (IPSS) over 19, classical resection was performed in 46 cases, “cold-knife" TUI in 37 cases and bipolar vaporization in 43 patients. The evaluation protocol comprised IPSS, QoL (quality of life) score, Qmax and PVR (post-voiding residual urinary volume) assessment performed at 1, 3, 6, 12, 18 and 24 months after the initial intervention.

Results: Significant intraoperative complications (capsular perforation – 8.7%; bleeding – 4.3%) occurred secondary to monopolar resection. “Star" TUI was the fastest technique, followed by plasma-button vaporization (7.2 and 11.4 versus 16.5 minutes). BPV and TUI patients benefitted from the shortest catheterization periods (0.75 and 1 versus 2.0 days) and hospital stays (1.0 and 1.25 versus 2.0 days). Immediate postoperative adverse events consisted of hematuria (6.5% of the TUR cases) and acute urinary retention (8.1% of the TUI group). Significantly higher long term BNS recurrence rates requiring re-treatment were established in the TUI (18.7%) and TUR (12.8%) series by comparison to BPV (5.4%). Among patients that completed the follow-up protocol, equivalent IPSS, QoL, Qmax and PVR features were determined in the 3 study arms.

Conclusions: The plasma vaporization approach was confirmed as a successful match to conventional TUR and “cold-knife" TUI in terms of surgical safety profile, postoperative recovery, therapeutic durability and urodynamic and symptom score parameters.

## Introduction

Despite the constant technological advances achieved in the lower urinary tract pathology various endoscopic modalities, obstructive late complications such as urethral strictures and bladder neck sclerosis (BNS) continue to represent relatively often encountered undesired events [**1**]. Additionally, open adenomectomy for benign prostatic hyperplasia (BPH) [**2**] and radical prostatectomy in prostate cancer cases [**3**] also displayed secondary BNS during the short and medium term postoperative evolution of these particular categories of patients. 

 Several therapeutic alternatives were described as possible solutions for this type of complication, the most popular techniques being constituted by monopolar transurethral resection (TUR) [**4**] and “cold-knife" transurethral incision (TUI) [**5**]. Regardless of the apparently easy technical process of relieving obstruction form the bladder neck area by either retrograde resection or incision, the literature data is still marked by several rather significant and persistent shortcomings [**6**]. 

 The presently available studies have been constantly characterized by the reduced number of cases included [**4**], insufficient follow-up periods [**7**], surprisingly disappointing follow-up outcomes [**8**] and disturbingly contradictory conclusions as to the most effective treatment approach [8,9]. Basically, the most frequently established drawback related to secondary BNS endoscopic surgery is represented by the insufficiently satisfactory capacity of these patients to maintain spontaneous voiding after surgery due to the unacceptably high sclerotic process’ recurrence rates [**6**].

 From another perspective, during the past several years, the bipolar plasma vaporization (BPV) was certified as a remarkably efficient therapeutic alternative in average size BPH cases, apparently even able to overcome the so far “gold-standard" conventional resection in terms of follow-up symptom scores and voiding parameters [**10**]. Based on these premises, BPV was introduced as a viable technique in secondary BNS pathology, initially enjoying superior short term clinical results [**11**].

 The present analysis was aimed to synthesize a single center experience in this yet improperly assessed field of endourology constituted by the secondary BNS’ minimally invasive treatment. A critical comparison of the most popular 3 such procedures was retrospectively achieved in light of a rather substantial series of patients.

## Material and method

A total of 126 BNS cases were included in this retrospective trial and followed during an average period of 24 months after the endoscopic treatment. Among this series, the bladder neck sclerotic obstruction occurred secondary to TURP in 84 cases, to open adenomectomy in 33 patients and to radical prostatectomy in 9 cases. 

 As far as the inclusion criteria were concerned, patients involved in this analysis presented severe lower urinary tract symptoms confirmed by an International Prostate Symptom Score (IPSS) over 19 and a maximum urinary flow rate (Qmax) below 10 mL/s. Concerning the follow-up evaluation protocol, all cases were assessed at 1, 3, 6, 12, 18 and 24 months after the initial procedure by IPSS, quality of life score (QoL), Qmax and post-voiding residual urinary volume (PVR). 

**Fig. 1:**
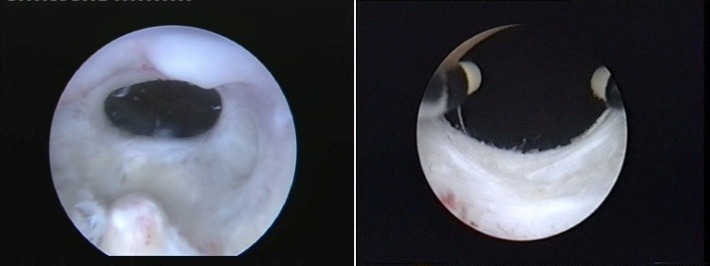
Monopolar circumferential TUR of the sclerotic tissue

 Conventional TUR was applied in 46 patients using the 26F Storz (Karl Storz, Tuttlingen, Germany) continuous flow monopolar resectoscope and single wire resection loops. The technique was practically similar to that of classical TURP, residing mainly in the circumferential resection of the fibrous tissue occupying the bladder neck (**Fig. 1**). 

**Fig. 2:**
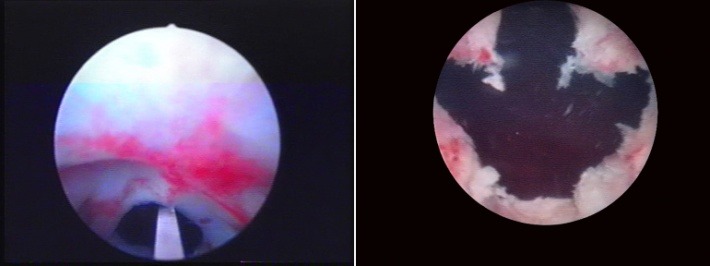
“Star" “cold-knife" incision of the bladder neck obstructive block

 Simple TUI was achieved in 37 patients by “cold-knife" “star" incision at the 12, 3, 6 and 9 o’clock positions of the bladder neck. For this technique, a 21F Storz urethrotome was used for obtaining deep incisions of the sclerotic block (**Fig. 2**)

**Fig. 3:**
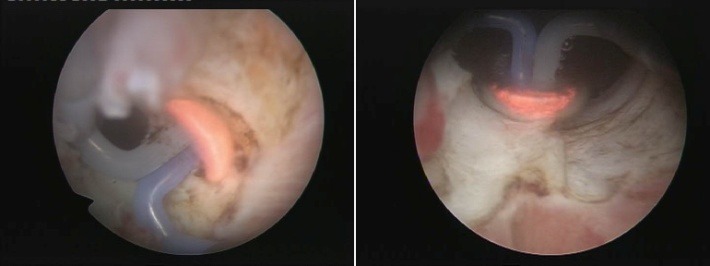
Bipolar plasma vaporization of the fibrous formation

**Fig. 4:**
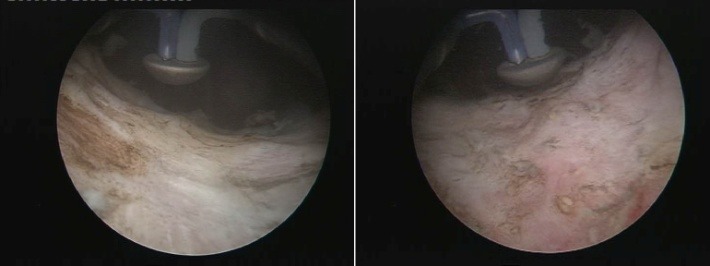
Well visible smooth surface of the prostatic capsule, cleared from any residual scarring tissue

**Fig. 5:**
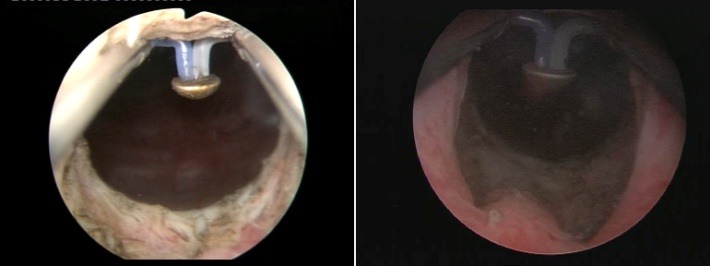
Obstruction-free prostatic fossa and bladder neck at the end of the procedure

The BPV procedure was performed in 43 cases, the technical setup being represented by the Olympus SurgMaster UES-40 bipolar generator, the OES-Pro bipolar resectoscope and “button" type vaporization electrodes (Olympus Europe, Hamburg, Germany). Regarding the actual surgical technique, the sclerotic formation was gradually ablated, layer by layer (the so-called “hovering" procedure) until reaching the prostatic capsule (**Fig. 3**). From the perspective of simple endoscopic observation, the complete ablation of the scarring tissue was certified by the clear image of clean capsular muscular fibers revealing a remarkably smooth and neat surface (**Fig. 4**) as well as by the open-wide prostatic fossa and bladder neck lacking virtually any kind of residual irregularities, debris or obstruction (**Fig. 5**).

 The main target of the study was to establish eventually significant differences between the 3 methods regarding the surgical safety and efficiency profile, perioperative features and follow-up urodynamic and symptomatic parameters.

## Results

From the point of view of the preoperative clinical assessment, similar determinations were established concerning the mean IPSS, QoL, Qmax and PVR values characterizing the respective study arms (**Table 1**).

**Table 1:** Preoperative features

Preoperative features	BPV (N=43)	TUR (N=46)	TUI (N=37)
IPSS	22.8	23.1	23.2
QoL	4.1	4.3	3.9
Qmax (mL/s)	8.0	7.4	7.9
PVR (mL)	124.7	113.5	118.6

The 3 techniques were successfully carried out in all the included patients. During conventional TUR, certain adverse events such as prostatic capsule perforation (8.7%) and intraoperative bleeding (4.3%) impeded on the accuracy of the fibrotic tissue removal (**Table 2**). In a single TUI case (2.7%), the deep scar incision was associated with a significant hemorrhage. The BPV procedure was not affected at any point during surgery by either type of complication. 

**Table 2:** Perioperative parameters

Perioperative parameters	BPV (N=43)	TUR (N=46)	TUI (N=37)
Operation time (minutes)	11.4	16.5	7.2
Capsular perforation	0%	8.7%	0%
Intraoperative bleeding	0%	4.3%	2.7%
Catheterization period (days)	0.75	2.0	1.0
Hospital stay (days)	1.0	3.0	1.25

 From the perspective of the mean operation time, the “cold-knife" incision constituted the fastest therapeutic approach (7.2 minutes), followed by the plasma-button vaporization (11.4 minutes). Due to the increased coagulation requirements and resection chips’ extraction, monopolar TUR displayed the longest average duration (16.5 minutes). As far as the postoperative recovery was concerned, by comparison to the clearly more invasive classical resection, the BPV and TUI techniques implied substantially decreased mean catheterization periods (2.0 versus 0.75 and 1 days, respectively) and hospital stays (3.0 versus 1.0 and 1.25 days) (**Table 2**).

 With regard to the immediate follow-up, the postoperative hematuria was only encountered in the TUR group (6.5%), while the re-catheterization due to acute urinary retention subsequent to catheter removal was present in 8.1% of the “star" incision patients and in a single standard resection case (2.2%). The early irritative symptoms’ profile emphasized a slightly increased frequency among the BPV and TUR series (13.9% and 10.9%) when compared to TUI cases (5.4%). No substantial differences were determined between the 3 study arms concerning the urinary tract infection rates (4.6% versus 4.3% and 2.7% for BPV, TUR and TUI, respectively) (**Table 3**).

**Table 3:** Early postoperative complications

Postoperative complications	BPV (N=43)	TUR (N=46)	TUI (N=37)
Postoperative hematuria	0%	6.5%	0%
Re-catheterization rate	0%	2.2%	8.1%
Early iritative symptoms	13.9%	10.9%	5.4%
Urinary tract infections	4.6%	4.3%	2.7%

 The 2 year’ dropout rates in the 3 above mentioned groups were 13.9%, 15.2% and 13.5%, respectively. Consequently, a total of 37, 39 and 32 patients of the BPV, TUR and TUI groups completed the entire 24 months’ follow-up protocol. While specifically referring to these thoroughly evaluated cases, at the 1, 3, 6, 12, 18 and 24 months’ check-ups, similar findings were determined regarding the mean IPSS, QoL, Qmax and PVR features in the BPV, TUR and TUI series, with no important differences between them whatsoever at any time during follow-up (**Table 4**). 

**Table 4:** Long term follow-up characteristics

Results	BPV (N=43)	TUR (N=46)	TUI (N=37)
IPSS			
1 month	4.6	4.4	4.7
3 months	3.9	4.2	4.3
6 months	3.7	3.5	4.0
12 months	3.2	3.6	4.1
18 months	3.5	3.3	3.8
24 months	3.1	3.2	3.5
QoL			
1 month	1.5	1.6	1.4
3 months	1.3	1.2	1.3
6 months	1.2	1.2	1.1
12 months	1.2	1.0	1.2
18 months	1.0	1.1	1.0
24 months	1.1	1.2	1.1
Qmax (mL/s)			
1 month	23.5	24.1	23.3
3 months	23.9	24.2	23.6
6 months	23.4	23.6	24.0
12 months	23.0	23.7	23.5
18 months	23.2	23.4	23.1
24 months	23.1	23.3	23.2
PVR (mL)			
1 month	39	37	48
3 months	32	34	43
6 months	36	30	32
12 months	27	34	37
18 months	24	38	35
24 months	31	35	38

 Additionally, the series in question displayed a particular category of cases which failed to maintain spontaneous voiding or presented a substantial decline in their urodynamic and symptom score parameters at some point during the prolonged evaluation. Such patients underwent endoscopic re-intervention aimed to restore their normal voiding capacity. Based on these data, the BNS long term recurrence rate and consequent re-intervention requirement was substantially reduced in the plasma-button vaporization series by comparison to the conventional transurethral resection or incision study groups (5.4% versus 12.8% and 18.7%, respectively).

## Discussion

While considering the available literature on the subject of bladder neck contracture secondary to prostate surgery, certain aspects are quite obvious. The published studies regularly display a shortage in patients’ numbers, reduced evaluation periods and both insufficient as well as disappointing follow-up features. Additionally, although several therapeutic alternatives were occasionally analyzed as possibly successful means of relieving BNS related obstruction, none of them managed to acquire a “standard treatment" status for this particular type of pathology. 

Contradictory reports supporting the various endoscopic techniques are in no position so far as to outline a long term feasible solution in such cases. In any case, since the BNS condition occurring subsequent to transurethral prostate surgery is consistently acknowledged as a significant urological daily practice problem [**19**], the present study was intended to bring further clarification of the most indicated treatment approach while also considering the relatively innovative technique of scar tissue plasma vaporization.

 To start with, transurethral incision of the bladder neck was often described as a somewhat efficient secondary BNS approach, although requiring a repeated intervention in part of the patients [**12**]. Further along this line, in cases of bladder neck contracture subsequent to radical prostatectomy, quite variable outcomes in terms of recurrence rates (9-38%) were determined as following the TUI treatment [6,13]. On the other hand, a more optimistic perspective on recurrent BNS management and prevention by radial retrograde incision seemed to be offered by the association of intralesional injection of the antiproliferative mitomycin C agent (72% overall success rate after 1 procedure and 89% after 2 interventions) [**14**]. Early studies underlined superior outcomes of the “cold-knife" incision technique, as shown by the up to 90% immediate success rate as well as the satisfactorily good long term evolution (mean 44.5 months) of these patients [**20**].

 Although certain reviews underlined “cold-knife" incision as first-line treatment [**15**], other trials characterized TUI as achieving the least satisfactory outcomes (75% recurrence prognosis), while the sclerotic tissue ablation by transurethral encircling resection of the obstructive diaphragm was found as the most widely used therapeutic solution that is also in the position to provide better results [**16**]. Following this perspective, monopolar TUR removing the anastomotic stricture secondary to radical prostatectomy was shown as a preferable solution when drawing a parallel to the simple endoscopic incision due to the generous tissue resection that it enables [**17**]. 

 A confirmation of this therapeutic principle implying scar block complete removal is represented by the significantly reduced recurrence rates after conventional TUR when compared to radial retrograde incision (22.2% versus 38.5%) [**8**]. Further more, the classical resection displayed better outcomes with regard to the category of patients that emphasized unsatisfactory short term results. As of such, a substantially lower rate of cases characterized by a poor early postoperative evolution was described as subsequent to TUR by comparison to bladder neck incision (26.6% [**18**] versus 36% [**16**]).

 When considering the data acquired during the course of the present retrospective analysis, the plasma-button vaporization could certainly be underlined as a correct answer to the perpetual BNS undesired evolution issue. Promisingly enough, the BPV technique managed to provide a substantially decreased long term bladder neck contracture’ recurrence rate by comparison to TUR and TUI (5.4% versus 12.8% and 18.7%, respectively). Further more, these results confirm the above mentioned idea that the fibrous tissue radical ablation by either vaporization or resection is significantly more efficient than the simple retrograde incision leaving behind the entire scarring block in place and therefore susceptible to eventual recurrences. 

 Most importantly, the 2 year’ follow-up period implied by this trial could be considered as optimistically confirming the plasma vaporization as an effective long term solution, particularly since previous studies outlined a mean period of 9 months’ delay (ranging between 3 and 15 months) from the initial intervention for the occurrence of bladder neck contracture’ recurrence [**8**].

 At this point, it may be useful to mention the holmium yttrium aluminum garnet (Ho:YAG) as another possible alternative for BNS pathology, especially in cases of strictures affecting the vesicourethral anastomosis after radical prostatectomy. Usually in this particular field, “cold-knife" incision is still acknowledged as the most frequently used procedure [15,21]. However, although no cohort or comparable randomized multicenter trials yet exist concerning this relatively attractive technical modality, Ho:YAG incision is regarded as a safe and at least minimally invasive second-line treatment modality, profiting from the superior cutting properties of the laser which include minimal blood loss and less induction of scar tissue formation [**21**].

 A remarkably relevant aspect in BNS treatment is constituted by the virtually similar principle of fibrotic tissue removal reuniting the plasma-button vaporization and Ho:YAG incision. More precisely, after initially carrying out a deep incision of the obstructive scar formation, a vaporizing resection of the residual sclerotic tissue is recommended as part of the holmium laser approach, while aiming to achieve a complete scar ablation reaching down to the well-vascularized surrounding tissue layers [**22**].

 Going by the same concept, it may be interesting to discuss the predecessor of the plasma-button technique, namely the PlasmaKineticTM bipolar vaporization. More or less the same as BPV, this apparently safe, inexpensive and reliable therapeutic option was described by some studies as featuring minimal surgical morbidity and negligible blood loss [**23**]. From a different perspective related to the actual evidence based treatment outcome, the one year’ overall success rate (60%) and mean Qmax (16.2 mL/s) shown by the initial series [**23**] were substantially less satisfactory when compared to the plasma vaporization results (94.6% and 23.0 mL/s, respectively). Additionally, with regard to the perioperative features, both types of vaporization displayed superior postoperative recovery capabilities, as confirmed by the practically similar and quite short catheterization periods (1.0 [**23**] and 0.75 days). 

 Further along the path of clinical outcomes, a parallel with another modality of achieving the bladder neck incision seems to be in order, namely the 70-W 2-micron continuous wave laser (RevoLix, LISA laser products, Katlenburg, Germany). Basically, when compared to the plasma-button vaporization, at the 3 and 12 months’ check-ups, this type of laser TUI emphasized resembling Qmax values (25 versus 23.9 and 23 versus 23 mL/s), somewhat inferior IPSS (8 versus 3.9 and 8 versus 3.2) and practically similar QoL scores (1 versus 1.3 and 1 versus 1.2) [**24**]. Maybe relevant enough, the restenosis rate requiring re-treatment was increased among the thulium:YAG patients when facing a comparison to the BPV therapeutic approach (14.3% [**24**] versus 5.4%).

 From a subjective perspective and while considering the practical surgical features, certain explanations may be outlined for the superiority of the bipolar vaporization over both retrograde resection and TUI. As previously established, the simple bladder neck incision is marked by the substantial drawback of leaving the scar formation in place. Conventional single wire loop resection often constitutes a rather blunt tool while attempting to remove the usually thin but dense layers of sclerotic tissue. This kind of technique is many times exposed to prostatic capsule perforation, significant intraoperative bleeding and residual fibrous tissue. On the contrary, the BPV procedure creates the premises for a complete scarring block ablation in a gradual and well controlled manner, layer by layer, from the surface up to the healthy capsular fibers.

 Naturally, further long term, prospective, multicenter studies are required while aiming to come up with a definite solution for the still important prostate surgery shortcoming represented by the secondary bladder neck contracture. However, to say the least, the plasma vaporization modality appears to provide a promising alternative for this kind of pathology, susceptible of setting up a certain standard of treatment.

## Conclusions

The present analysis established a reliable evidence based progress in secondary BNS therapeutic management in light of the plasma-button vaporization innovative approach. The retrospective gathering of data from a rather extensive series of patients involving this particular lower urinary tract pathology confirmed the long term BPV efficacy as a successful continuation of the initial surgical advancement.

 The conventional monopolar TUR was marked by significantly higher perioperative bleeding risks, longer operation time and increased prostatic capsule perforation frequency. On the other hand, the “cold knife" TUI series displayed substantially shorter operation time and a decreased early irritative symptoms’ rate but suffered from an elevated immediate postoperative re-catheterization hazard caused by acute urinary retention (otherwise also present among the classical resection patients).

 From the perspective of the postoperative recovery abilities provided by the 3 types of procedure, both BPV and TUI created the premises for a faster convalescence period to be achieved, as confirmed by the significantly decreased catheterization period and hospital stay. With regard to the actual surgical quality, the bipolar vaporization procedure emphasized remote hemorrhagic and perforation risks, satisfactory sclerotic tissue ablation speed and irritative symptoms’ profile as well as superior spontaneous voiding capacity subsequent to catheter removal upon discharge.

 During the 2 year’ follow-up, the regular check-ups determined similar urodynamic and symptom score features characterizing patients from the 3 study arms that were able to complete the entire evaluation protocol. A remarkable therapeutic advance offered by the plasma-button technique was represented by the substantially reduced BNS long term recurrence rate and re-treatment necessity, thus proving the reliable durability of the fibrous block complete ablation phenomenon.
